# Endoscopic Sphenopalatine Artery Cauterization in the Management of Recurrent Posterior Epistaxis

**DOI:** 10.3390/medicina59061128

**Published:** 2023-06-12

**Authors:** Sever Septimiu Pop, Cristina Tiple, Mirela Cristina Stamate, Magdalena Chirila

**Affiliations:** 1 ENT Department, “Iuliu Hatieganu” University of Medicine and Pharmacy, 400006 Cluj-Napoca, Romania; severpop@me.com (S.S.P.); cristinatiple@yahoo.com (C.T.); mctmedic@yahoo.com (M.C.S.); 2 ENT Clinic, Emergency County Hospital, 400006 Cluj-Napoca, Romania

**Keywords:** sphenopalatine artery, endoscopic, cauterization

## Abstract

*Background and Objectives*: Endoscopic sphenopalatine artery cauterization (ESPAC) has become a reliable and effective surgical procedure for managing posterior epistaxis. The objectives of our study were to evaluate the effectiveness of ESPAC in the management of posterior epistaxis and the possible factors that lead to the failure of the procedure. *Materials and Methods*: We performed a retrospective analysis of all patients who underwent ESPAC between 2018 and 2022. We retrospectively reviewed the demographic data, patients’ co-morbidities, medical treatment conditions, whether other surgical procedures were performed in addition to the ESPAC, and the success rate of ESPAC. *Results*: 28 patients were included in our study. After ESPAC, epistaxis was successfully managed in 25 patients (89.28%). Of all patients undergoing ESPAC, three (10.7%) presented re-bleeding. In two patients, we performed an endoscopic revision surgery with re-cauterization of the sphenopalatine foramen area, together with anterior and posterior ethmoidectomy, followed by fat occlusion/obliteration of these sinuses. In one patient, fat obliteration of the anterior and posterior ethmoid was also unsuccessful, and we performed an external carotid artery ligation at the level of the neck with no recurrence afterwards. *Conclusions*: Endoscopic cauterization of the sphenopalatine artery remains a safe, effective, and reliable surgical procedure in the management of recurrent posterior epistaxis. The use of anticoagulant drugs and the association of hypertension and other heart and liver diseases do not materialize as factors influencing surgical failure.

## 1. Introduction

Epistaxis represents one of the most frequent otolaryngological emergencies, affecting up to 60% of the population during their lifetime, with 6% requiring medical intervention [1]. There are two incidence peaks, one in patients aged below 10 years and the other in patients above 40 years [2].

Epistaxis is classified as anterior epistaxis when the bleeding source is located anterior to the plane of the pyriform aperture and posterior epistaxis [3]. Many patients are managed conservatively, using various types of nasal packings (anterior, posterior, with Vaseline gauze, expandable Merocel, and balloon catheters). However, recurrent posterior epistaxis continues to be a challenge, even for the most experienced otolaryngologists. 

Endoscopic sphenopalatine artery ligation (ESPAL) or cauterization (ESPAC) has become a reliable and effective surgical procedure for managing these patients. It successfully replaces the need for repeated posterior packings and significantly decreases hospitalization and treatment costs.

The objectives of our study were to evaluate the effectiveness of ESPAC in the management of posterior epistaxis and the possible factors that lead to the failure of the procedure.

## 2. Materials and Methods

We performed a retrospective analysis of all patients who underwent endoscopic sphenopalatine artery cauterization (ESPAC) in our department between 2018 and 2022. The main inclusion criteria were a history of severe posterior epistaxis requiring hospitalization and the failure of multiple anterior and posterior packings. Patients with epistaxis secondary to trauma, post-surgical epistaxis, and epistaxis secondary to benign or malignant tumors were excluded from the study.

We retrospectively reviewed the demographic data, patients’ co-morbidities, medical treatment conditions, whether or not other surgical procedures were performed in addition to the ESPAC, and the success rate of ESPAC.

The following variables were evaluated: gender, smoking habits, the associated presence of hypertension, other cardiovascular diseases, diabetes, liver diseases, and the use of anticoagulant drugs.

The procedure was performed in all patients under general anesthesia with the patient in a reverse Trendelenburg position with at least 15° of head elevation, which reduces mucosal bleeding during surgery [4]. After the packing extraction, a careful toilette of the nasal cavity with the removal of the blood clots was performed, and nasal cavities were examined with a 0° endoscope. If not strongly contraindicated, a local infiltration with lidocaine-adrenaline (1:100,000—lidocaine) solution was applied at the level of the lateral nasal wall (middle turbinate insertion and the uncinate process). Pieces of cotton with topical vasoconstrictor were inserted in the nasal cavities before starting the procedure for at least five minutes. Medialization of the middle turbinate was sometimes required to get wide access to the middle meatus. If a septal deviation or a septal spur did not allow adequate access to the targeted zone, a septoplasty (either classic or endoscopic) was performed.

**Figure 1:**
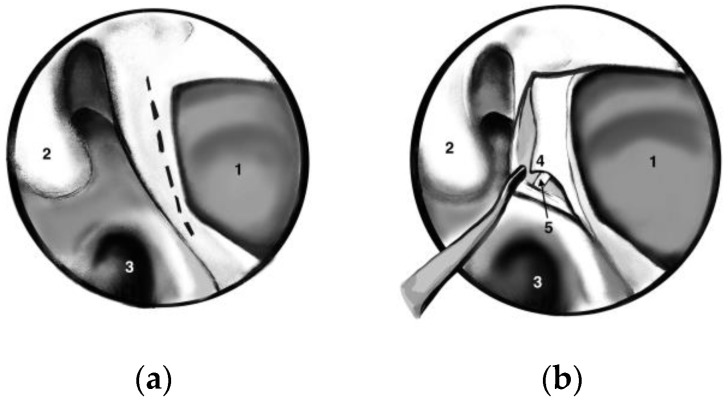
Endoscopic exposure of the left sphenopalatine artery. (**a**) The vertical incision of the mucosa (dot line), (**b**) the elevation of the mucosal flap with left sphenopalatine artery exposure (arrow). 1—antrostomy (posterior wall of the maxillary sinus), 2—middle turbinate, 3—choanae, 4—mucosal flap, 5—sphenopalatine artery.

A standard uncinectomy with identification of the natural osmium of the maxillary sinus was followed by a wide antrostomy that reached the posterior wall of the sinus. We performed a wide antrostomy with enlargement of the maxillary sinus ostium in all patients, even though many authors suggest that this antrostomy is not required in all cases. A vertical incision at this level with the elevation of the mucosal flap allowed the identification of the sphenopalatine artery (SPA) using all the anatomical landmarks (posterior wall of the antrum, middle turbinate root and, most important of all, crista ethmoidalis) as seen in Figure 1. Crista ethmoidalis is a small triangular projection of bone located anterior or anteroinferior from the sphenopalatine foramen, pointing medially to the nasal cavity. Its location is posterior to the posterior wall of the antrum, anterior to the vertical face of the sphenoid bone, superior to the inferior margin of the horizontal part of the middle turbinate, and inferior to the posterior ethmoidal cells [2]. The sphenopalatine foramen is found in an area within 1 mm of the crista ethmoidalis in 95% of patients [2].

Bipolar cauterization of all branches of the SPA was performed, followed by local placement of Surgicel, a resorbable hemostatic agent. Soft packing was removed after 24–48 h. Antibiotics and hemostatics were prescribed for 2–3 days. After that, the topical application of a nasal saline spray was recommended for at least two weeks. 

Statistical analysis was performed using Wizard 2 for Mac, version 2.0.13 (262) (Evan Miller, Chicago, IL, USA). Relationships between re-bleeding (revision surgery) and the aforementioned preoperative variables were analyzed (*p* < 0.05 was considered statistically significant).

## 3. Results

Twenty-eight patients were included in our study, aged between 43 and 73 years old, with a mean age of 60.821 and a standard deviation of 7.313. Of these, 18 were males (64.3%) and 10 were females (35.7%). Related to co-morbidities, 22 were diagnosed with arterial hypertension (78.6%), 17 with other heart diseases (60.7%), 6 with type II diabetes (21.4%), and 7 with liver diseases (25%). Sixteen patients (57.1%) were under anticoagulant treatment, and nineteen (67.9%) were smokers. The patients’ data are depicted in Table 1.

Anterior and posterior packing failed to control the bleeding in all patients. Six patients (21.4%) underwent one nasal packing before surgery, twenty-one patients (75%) underwent two packings, and one (3.6%) patient underwent three packings. Three patients (10,71%) required a concomitant septoplasty to allow adequate access to the targeted area.

After ESPAC, epistaxis was successfully managed in 25 patients (89.28%).

Of all patients undergoing ESPAC, three (10.7%) presented re-bleeding. In two patients we performed an endoscopic revision surgery with re-cauterization of the sphenopalatine foramen area, together with anterior and posterior ethmoidectomy, followed by fat obliteration of these sinuses. The adipose tissue was harvested from the level of the abdominal subcutaneous fat. In one patient, fat obliteration of the anterior and posterior ethmoid was also unsuccessful. Therefore, we performed an external carotid artery ligation at the level of the neck with no recurrence afterwards.

No intraoperative complications were reported. The only postoperative complications were minor: crusting and nasal dryness in three patients and ipsilateral palatal numbness in one patient. All complications were successfully managed conservatively.

The mean hospitalization was 7.86 days, ranging from 5 to 11 days, with a standard deviation of 1.46 days.

There was no statistically significant relationship between the need for revision surgery and smoking, hypertension, other heart diseases, liver diseases, and the treatment with anticoagulant drugs. A statistically significant correlation was noted between revision surgery requirement and the presence of diabetes (chi-square test, *p* = 0.043) and the number of nasal packings before surgery (chi-square test, *p* = 0.011).

## 4. Discussion

In most patients, anterior epistaxis does not require medical intervention. For those cases presenting at an ENT Department, the management is usually simple, using different types of anterior packing (Vaseline gauze, expandable Merocel, balloon catheters) or cauterization of the bleeding point if visible. In contrast, posterior epistaxis is more challenging, with a high incidence of recurrences after the initial treatment. Posterior nasal packing either with Vaseline gauze or nasal balloon catheters is often used but is not always effective. These patients undergo repeated unsuccessful posterior packings with prolonged hospitalization. It has been prospectively demonstrated that patients with posterior epistaxis are more susceptible to hospitalization, are twice as likely to require nasal packing, and a prolonged hospital stay is needed [5]. In such cases, more aggressive approaches such as surgery or embolization are essential [6]. A Swiss retrospective cohort study demonstrated that surgical hemostasis is significantly superior to packing in managing these patients, with a treatment failure rate of 3% vs. 38% [7].

Budrovich and Saetti [8] were the first to report trans-nasal endoscopic approaches to ligate the sphenopalatine artery (SPA) in 1992. Since then, the endoscopic approach has gained enormous popularity and has become a very effective modality for managing patients with intractable posterior epistaxis. Consequently, there is a trend in the medical literature to recommend ESPAL/ESPAC as a first-line approach in the management of these cases. In a prospective randomized trial published in 2004, Moshaver et al. demonstrated a significant reduction in cost and length of hospitalization of the patients undergoing endoscopic sphenopalatine artery ligation (ESPAL) compared with the conventional nasal packing [9]. Therefore, they recommended ESPAL as the first-line treatment in selected patients. Dedhia et al. also suggested that first-line ESPAL is superior to a 3-day posterior packing regarding cost-effectiveness [10]. Rudmik and Leung [11] suggested that when both ESPAL and embolization are viable options, ESPAL should be the first choice because it is more cost-effective.

Tessler et al. [12] reported their experience in managing persistent epistaxis during a 15-year period, dividing the patients into two groups: pre-ESPAL and post-ESPAL (related to the moment when ESPAL was adopted in their department). ESPAL incorporation resulted in a significant decline in the use of posterior nasal packings. The post-ESPAL group had lower rates of early postoperative re-bleeding when compared to the pre-ESPAL group (0.0% vs. 9.2%, *p*-value = 0.02). The “haemoglobin recovery levels” were also higher, and the one-year mortality rate was significantly lower in the post-ESPAL group.

Related to the surgical technique, we performed a wide antrostomy with enlargement of the maxillary sinus ostium to the level of the posterior sinus wall in all patients, even though many authors suggest that this antrostomy is not always required [13,14,15]. Moreover, Shires et al. [16] demonstrated in a cadaver anatomic study that 90% of ESPAL can be successfully performed using an isolated nasal wall incision without the need for a large antrostomy. However, we consider the posterior wall of the maxillary sinus to be a very reliable landmark, mainly in patients in whom the ethmoidal crest is difficult to find or when the posterior fontanelle cannot be accurately evaluated.

Most authors suggest that nasal packing is not necessary after ESPAC/ESPAL [2,4]. However, we used a soft non-absorbable nasal packing for 24–48 h in all patients for additional hemostasis due to nonarterial bleeding.

There are debates in the literature regarding the effectiveness of ligation vs. cauterization. However, in a study performed on 67 patients and published in 2007, Nouraei et al. concluded that cauterization is superior to ligation and that not using diathermy was an independent risk factor for failure of the procedure [17]. Furthermore, a systematic review published in 2019 [6] also demonstrated that sphenopalatine artery cauterization is more effective than ligation in treating refractory epistaxis. The pooled total re-bleeding rate of cauterization was 7.2% (95% CI 4.6–11), compared to 15.1% (95% CI, 9.8–22.5) for ligation procedures. Another advantage of cauterization should be a significantly shorter operative time, as reported by Chitsuthipakorn et al. in 2020 [18]. The most important parameter to assess the efficacy of this procedure is the re-bleeding rate.

In our study, the re-bleeding rate was 10.7%, similar to data reported in the medical literature. In a recent systematic review published in 2019 [6], including 33 papers and 896 cases of ESPAL and ESPAC, the pooled total re-bleeding rate for sphenopalatine surgery was 13.4% (95% confidence interval (CI), 10.0–17.8%). The re-bleeding rate for selected treatments in included studies ranged from 0% to 30%.

Anatomical variations represent an essential cause for re-bleeding after SPA surgery. Padua and Voegels [19] reported only a single branch emerging through the sphenopalatine foramen in most cadaver nasal cavities (67.21%), followed by two (21.31%) and three (11.47%) branches. On the other hand, there are authors reporting two branches in most cases [20,21]. Overall, the number of SPA branches described in the literature varies from one to ten [22]. If the cauterization is performed only on one branch in patients with multiple branches, this could be a reason for re-bleeding [6]. In our opinion, cauterization is superior to ligation, mainly in these cases with multiple branches, because the cauterization of a larger area around the sphenopalatine artery allows the inclusion of as many branches as possible. After cauterization, we also applied some resorbable hemostatic agent (Surgicel) on the entire area of the sphenopalatine foramen.

To decrease the risk of re-bleeding, Saraceni et al. [23] recommend the resection of the ethmoidal crest during the surgical procedure. This provides better exposure to the SPA, allowing a more precise cauterization of all branches. Piastro et al. reported in 2018 a dual sphenopalatine artery and internal maxillary artery approach, laterally within the pterygopalatine fossa, providing near complete control of bleeding from the sphenopalatine area [24].

In our study, no significant correlations were found between the need for revision surgery and smoking, hypertension, other heart diseases, liver diseases, and the treatment with anticoagulant drugs. This is in correlation with other data published in the medical literature [14]. The only significant perioperative factors were the presence of diabetes and the number of preoperative posterior packings. Nouraei et al. [17] found that the administration of warfarin, not using diathermy for SPA occlusion, and platelet count on admission were significant factors for re-bleeding on univariate analysis. Platelet counts on admission and not using diathermy were independent and significant factors in multivariate analysis. The relationship between hypertension and epistaxis is still a controversial topic. Elevated blood pressure is noticed in nearly all patients with epistaxis, but if hypertension is the bleeding cause or is a result of the anxiety induced by hospital admission and the invasive procedures to control the bleeding is not demonstrated [3].

The anterior ethmoid artery is an unusual bleeding source in epistaxis. It is typically responsible for nasal bleeding in skull base fractures secondary to trauma or in iatrogenic injuries (endoscopic sinus surgery and skull base surgery). The anterior ethmoid artery ligation can be performed endoscopically or using an open transorbital approach [2]. The endoscopic approach involves a complete ethmoidectomy with the identification and exposure of the skull base [25,26]. The role of anterior ethmoid artery ligation in conjunction with ESPAL/ESPAC is controversial. However, there is evidence suggesting that this association of procedures can be beneficial for some patients [6]. In our study, we successfully performed an endoscopic fat obliteration of the anterior ethmoid in two patients with relapse after ESPAC. Therefore, this could be a viable option in cases where the anterior ethmoid artery ligation is challenging to perform. 

None of the patients from our study reported significant complications. One possible disadvantage of ESPAC could be the proximity of the cauterization area to the pterygopalatine fossa, which contains some important neural structures. The dissemination of thermal or electrical energy could potentially damage these structures [18]. Only one patient in our study presented ipsilateral palatal numbness postoperatively, but this complication resolved spontaneously. 

The complication rate is also very low in the medical literature. For example, no major complications were reported in a retrospective review of 38 patients published by Snyderman et al. in 1999 [27]. Minor complications included nasal crusting, teeth, palate, and upper lip numbness, and acute sinusitis. Other minor complications reported are synechia formation, septal perforation, and xerophthalmia. A systematic review published in 2019 by Kitamura et al. [6] reported a pooled perioperative complication rate of 8.7% (95% CI, 4.9–15.1). There was no significant difference between the cauterization group and the ligation group (10.2% vs. 6.4%). Among the 31 enrolled studies, complications were reported in 14 studies, ranging from 0% to 27%. The most frequent complication was nasal crusting (8–21%), followed by acute sinusitis and nasal dryness (10%). Moorthy et al. reported in 2003 a case of inferior turbinate necrosis following endoscopic sphenopalatine artery ligation in a patient who underwent multiple packings before surgery [28]. In addition, one Brazilian retrospective longitudinal study reported a case of amaurosis after the intervention [29].

The mean hospitalization in our study was 7.86 days, ranging from 5 to 11 days, with a standard deviation of 1.46 days. These values are relatively higher compared to other data from the literature. The main reason could be that patients with severe comorbidities and severe epistaxis undergoing a posterior packing in an emergency setting are usually instantly admitted to the hospital in our department. Therefore, the surgical approach is indicated in hospitalized patients only after the failure of the posterior packing and sometimes after the failure of two consecutive posterior packings. Most of the hospital stay in our study was a result of conservative treatment attempts before surgery was performed. Minni et al. [30] reported a mean hospital stay of 2.96 days. The study included patients with at least one nasal packing in the three weeks before hospital admission. Therefore, patients were admitted to the hospital only for the endoscopic surgical procedure. Saraceni et al. [29] also reported a mean hospitalization of 3.38 days with a standard deviation of 4.53 days in a study performed on 98 patients. Nouaraei et al. [17] reported an average length of postoperative patient stay of 2.6 ± 3.9 days and a total length of patient stay of 6.1 ± 5.9 days, similar to our results.

Other options for the surgical management of intractable posterior epistaxis are external carotid artery and internal maxillary artery ligation. External carotid artery ligation requires a neck incision and poses the risk of damaging important structures such as the vagus and the hypoglossal nerves [31]. We used the external carotid artery ligation in one patient as an adjunct procedure to control a re-bleeding after SPA cauterization. 

The internal maxillary artery ligation classically required a Caldwell–Luc approach, which could also lead to several complications such as sinusitis, infraorbital nerve damage, oro-antral fistula, dental injury, and blindness [31]. Nowadays, the internal maxillary artery can be approached endoscopically through the nose. The exposure of the vessel requires a wide maxillary antrostomy with visualization of the posterior wall of the maxillary sinus. A drill, a Kerrison or a Citelli punch forceps removes part of the posterior bony wall. The outer periosteum is incised using an electrocautery, and the fat of the pterygopalatine fossa is exposed. The internal maxillary artery is identified as a pulsatile structure, isolated, and ligated. There is a potential risk of damaging important nervous elements such as the vidian nerve, the greater palatine nerve, and the infraorbital nerve with side effects including decreased lacrimation, palatal, nasal, and cheek paresthesias [2]. The reported results of internal maxillary artery ligation are similar to the ESPAL/ESPAC, although this procedure is mainly used in skull base procedures such as transpterygoid approaches and resection of juvenile nasopharyngeal angiofibroma [2,24,32].

Embolization is another important option in the management of recurrent posterior epistaxis, but it requires an experienced invasive neuroradiologist. The main vascular targets in embolization for epistaxis are the internal maxillary and the sphenopalatine artery branches. The embolization of the internal carotid system, including the anterior and posterior ethmoidal arteries, is contraindicated due to possible severe complications such as blindness or stroke. A systematic review published in 2017 [33] found similar positive results between embolization and ESPAL/ESPAC (75–92% vs. 73–100%, respectively), although embolization was associated with more severe side effects (stroke was reported in 1.1–1.5% of the cases). Thirty-seven studies were identified related to surgical treatment, and 34 articles related to interventional radiology. No articles directly compared the two techniques. There is also a risk in embolization for systemic complications such as inhalation hypoxia, hypovolemia, angina, and myocardial infarction [34,35]. However, recent technological advances have led to a decrease in the complication rate and an augmentation of the success rates [6].

Related to technical advances, Karkos and Stavrakas [36] recently reported the use of a flexible 980 nm diode laser fiber to coagulate the SPA branches in cases when access to the nose was difficult secondary to unfavorable anatomy (septal spurs and narrow nasal cavities).

Our study has some limitations, being retrospective and performed on a relatively small number of patients. Therefore, further prospective, randomized studies are required to consolidate the primary role that should be played by ESPAC/ESPAL in the management of patients with recurrent posterior epistaxis.

## 5. Conclusions

Endoscopic cauterization of the sphenopalatine artery remains a safe, effective, and reliable surgicaSl procedure in the management of recurrent posterior epistaxis. The use of anticoagulant drugs and the association of hypertension and other heart and liver diseases do not materialize as factors influencing surgical failure. The presence of diabetes and the number of preoperative posterior packings seem to correlate significantly with recurrences after ESPAC.

## Figures and Tables

**Table 1:** Patient demographics.

	*N*	%
***N***	28	
Sex		
males	18	64%
females	10	36%
Smokers		
yes	19	67.85%
no	8	32.15%
HTA		
yes	22	78.57%
no	6	21.43%
Other heart diseases		
yes	17	60.71%
no	11	39.29%
Diabetes		
yes	6	21.43%
no	22	78.57%
Liver diseases		
yes	7	25%
no	21	75%
Anticoagulants		
yes	16	57.14%
no	12	42.86%

HTA—arterial hypertension.

## Data Availability

The data presented in this study are available on request from the corresponding author. The data are not publicly available due to privacy and ethical reasons.
